# Imatinib-induced Ototoxicity in a Patient with Gastrointestinal Stromal Tumor (GIST)

**DOI:** 10.7759/cureus.848

**Published:** 2016-10-26

**Authors:** Komal Wasif, Nawal Wasif, Muhammad W Saif

**Affiliations:** 1 Brookline High School, Tufts Medical Center; 2 Hematology/Oncology, Tufts Medical Center, Tufts University School of Medicine

**Keywords:** gastrointestinal stromal tumors, imatinib, deafness, tinnitus

## Abstract

Imatinib (Gleevec) is a biological agent that is approved for the treatment of chronic myeloid leukemia (CML) as well as gastrointestinal stromal tumor (GIST). The most frequently seen adverse effects in patients treated with imatinib include superficial edema, muscle cramps, musculoskeletal pain, rash, fatigue, headache, abdominal pain, and joint pain. Ototoxicity has rarely been reported except in two cases. We report a case of bilateral irreversible sensorineural hearing loss (SNHL) caused by imatinib in a patient receiving this agent in the adjuvant setting. This case underlines the importance of early recognition of this potential toxicity that can impact the quality of life.

## Introduction

Imatinib (Glivec or Gleevec) entered the oncology world with a bang and is considered as a miracle drug. Imatinib is a 2-phenylaminopyrimidine compound that specifically interacts with the adenosine triphosphate (ATP) binding site of multiple tyrosine kinases (TK) including BCR-ABL (an aberrant tyrosine kinase resulting from a fusion protein product of the acquired Philadelphia chromosome identified in greater than 90% of patients with CML), ABL-related gene product (ARG), and certain subgroup III receptor tyrosine kinases (c-kit receptor, platelet-derived growth factor (PDGF) receptor, and stem cell factor receptor) [1]. In May 2001, imatinib was approved by the Food and Drug Administration (FDA) for the treatment of patients with CML and later approved for the treatment of unresectable and/or metastatic malignant GISTs that express the tyrosine kinase receptor c-kit [2-3]. After an extensive experience with this agent in oncology and non-oncology fields, there is better understanding of both the short and long-term toxicities of the agent. The most common adverse effects encountered in patients treated with imatinib include superficial edema, muscle cramps, musculoskeletal pain, rash, fatigue, headache, gastrointestinal side effects including abdominal pain, and joint pain in 10% of the cases. Less frequent side effects with reported incidence of one to 10% include pancytopenia, febrile neutropenia, flushing, and liver function test abnormalities. Secondary malignancies, Sweet’s syndrome, angioedema, cardiac arrest and other rare toxicities have been seen in less than one percent of the patients.

Imatinib is considered to be a highly selective TK inhibitor (TKI), but it is expected that such TKIs can produce untoward toxicities not only on the target but also on non-target TKs. Imatinib-induced ototoxicity such as sensorineural hearing loss has been reported in a total of two cases treated with imatinib: in one patient with nephrogenic systemic fibrosis (NSF) and in another patient with CML respectively [4-5]. We report here the case of a patient who developed ototoxicity consisting of deafness, tinnitus, and vertigo secondary to use of imatinib for GIST. Informed consent was obtained from the patient for this study.

## Case presentation

The patient is a 61-year-old woman with a past medical history remarkable for rheumatoid arthritis, who presented to our institution with a rash 2 cm from the anal verge. Upon further questioning, the patient reported that she had leakage of a small amount of stool and also passing of a small amount of bright red blood in her stool over the past many months. On physical examination, a palpable mass was present just proximal to the anal verge. Imaging and a rectal endoscopic ultrasound (EUS) or echo-endoscopy with fine needle aspiration (FNA) biopsies were obtained, and the pathology was consistent with rectal gastrointestinal stromal tumor (GIST). She underwent neoadjuvant treatment with imatinib 400 mg daily, followed by a successful complete rectal resection of the tumor. After one year, the patient underwent a follow-up examination, and postsurgical imaging was ordered. It showed a possible residual disease. Therefore, it was decided to restart imatinib postoperatively on a dose of 400 mg daily. The patient developed mouth sores, worsening fatigue and appetite, as well as facial swelling, thought to be due to imatinib. She had her dose of medication reduced to 200 mg daily due to the side effects including rash, mucositis, fatigue, and diarrhea. Subsequently, she developed ill-marginated, erythematous, rough papules following imatinib on both of her legs (calves). The skin biopsy was consistent with actinic keratosis (AK), which is widely accepted as a precursor to squamous cell carcinoma (SCC) formation. Imatinib was eventually stopped due to her lower extremity rash and concern for possibilty of progression to SCC.

After three months of follow-up, a computed tomography (CT) scan showed increased asymmetric soft tissue thickening of the rectum with adjacent fat stranding worrisome for tumor recurrence. This was followed by a positron emission tomography (PET) scan, which showed no definite evidence of focal abnormal radiotracer uptake in the chest, abdomen, or pelvis, except mildly increased radiotracer uptake in the right anterolateral aspect of the rectum. Additionally, she underwent an endorectal ultrasound and no masses were found as biopsy was negative for malignancy. However, at this time the patient decided to resume Gleevec because of the risk of recurrence of GIST. Gleevec was started at a lowered dose of 100 mg orally. She tolerated Gleevec very well except for G1 nausea.

She returned for another follow-up in six weeks. At this visit, the patient reported dizziness described as motion sickness occurring daily and recurring episodes of true vertigo over the last four weeks. The episodes lasted from seconds to minutes, and were position-induced. The episodes increased in frequency and occurred 24/7. In addition, she also complained of tinnitus and impaired hearing, more in the right ear than in the left ear. Her past medical history was negative for any previous hearing loss, noise exposure, motion intolerance, migraines, frequent childhood ear infections, or ear surgery. She denied dizziness, syncope, headaches, and changes in vision. She stated that she had right otorrhea following a plane flight with upper respiratory tract infection (URI) in 2006. She denied any history of headaches, seizures, difficulty walking, difficulty with speech, stroke, transient ischemic attacks (TIA), memory loss, numbness/tingling, weakness, loss of coordination, or difficulty sleeping.

She migrated from Hong Kong in 1988. She has no children. She has no history of smoking or alcohol abuse. She works in the legal department at a bank. Her only medications included gabapentin 100 mg orally twice a day, Gleevec 100 mg daily, and Cymbalta 30 mg daily. Her father died at the age of 103, and he was healthy with no history of deafness or other otolaryngeal disease.

A physical examination showed no signs of acute distress; the patient communicated easily and understood our conversation with a normal quality of voice. Her ears exhibited normal pinnae bilaterally, normal ear canal bilaterally, normal tympanic membranes (TM) bilaterally, Rinne + bilaterally, and Weber midline. A nose examination showed dorsum straight, normal septum midline, turbinates normal, middle meati normal with mucosa normal. The nasopharynx and eustachian tube orifices (ETO) were normal bilaterally, with normal eustachian tube opening/closing, and normal mucosa. Adenoids were absent. Oral cavity/oropharynx exhibited lips normal, buccal mucosa normal, teeth normal, gingiva normal, floor of mouth normal, tongue normal, hard and soft palate normal, and tonsils 2+. The posterior pharynx had normal mucosa and no lesions were seen. The posterior pharyngeal wall was also normal. A fiberoptic examination of the larynx/hypopharynx showed normal base of tongue, normal supraglottis, normal arytenoids and normal esophageal inlet, with true vocal cords being normal with normal mobility, false vocal folds normal, post-cricoid region normal, piriform sinuses normal, hypopharangeal walls normal, and subglottis normal. A neck examination showed adenopathy, no masses, parotid glands normal, submandibular glands normal, laryngeal framework normal, and thyroid gland normal without enlargement, tenderness or masses. The neck muscles were normal, there was no temporomandibular joint (TMJ) or muscular tenderness. A head and face evaluation showed overall appearance with no sinus tenderness. A neurologic examination showed alert and oriented x 3, cranial nerves II-XII intact, facial strength House-Brackmann Facial Nerve Grading System HB Grade I, extraocular movements intact (EOMI), gaze alignment normal, head thrust without nystagmus, no spontaneous nystagmus, Romberg test negative, and Fukuda stepping test without rotation or drift.

**Figure 1:**
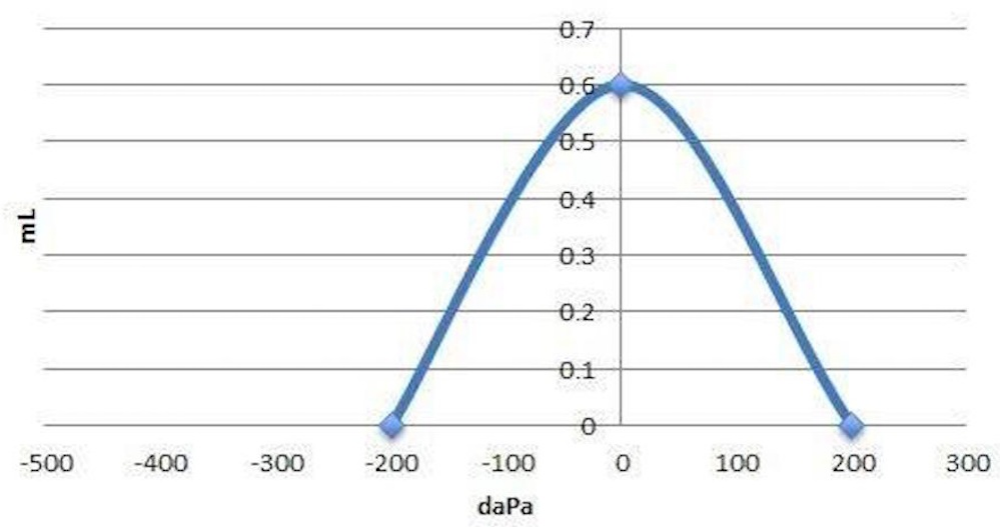
Videonystagmography (VNG) Videonystagmography (VNG) showed results within normal limits.

Videonystagmography (VNG) and video head impulse test (vHIT) were performed. Tympanometry was performed to verify middle ear status prior to caloric testing which showed normal middle ear functions bilaterally. An optokinetic test was attempted unsuccessfully as consistent recordings could not be obtained. A positional test revealed a two degree right-beating nystagmus in the head right, vision denied condition. These results were within normal VNG limits as shown in Figure 1.

**Figure 2:**
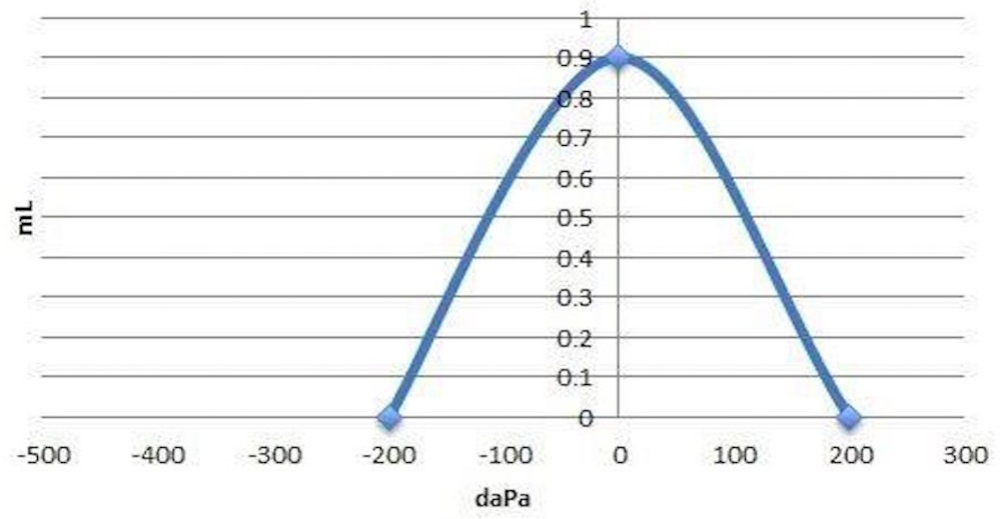
Video head impulse test (vHIT) Video head impulse test (vHIT) showed normal vestibular function of each semicircular canal.

The impulses were recorded using the ICS Impulse® system (GN Otometrics, Denmark). The test time was approximately 30 minutes. No significant overt or covert saccades were recorded. A reduced gain in the right anterior test was noticed, which could likely be due to camera interference with the patient's eyelashes. These results shown in Figure 2 were consistent with normal vestibular function of each semicircular canal.

**Figure 3:**
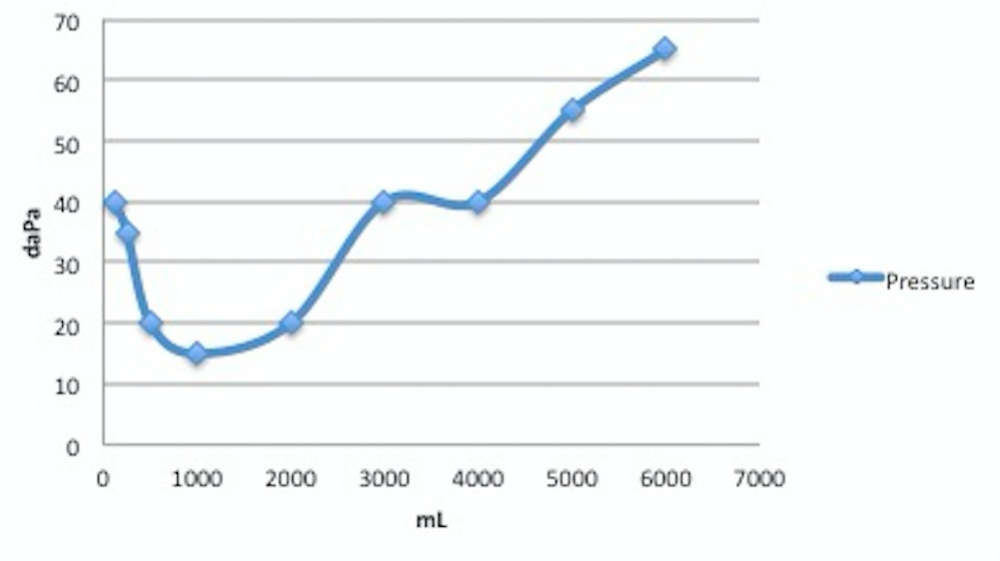
Audiogram of left ear Bilateral, asymmetric SNHL, right greater than left was found.

**Figure 4:**
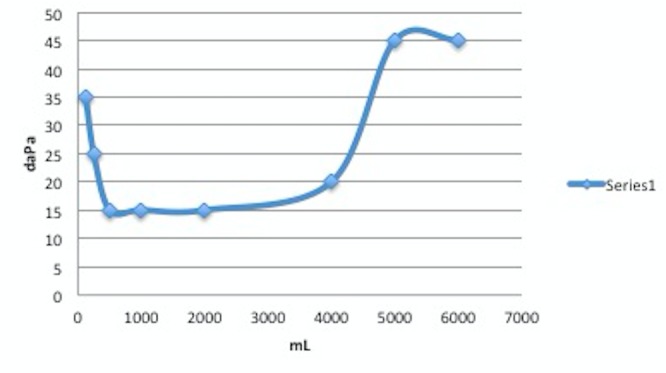
Audiogram of right ear Bilateral, asymmetric SNHL, right greater than left was found (series 1 of 2; the test was repeated twice).

An audiogram was performed. A mild low frequency SNHL rising to within normal limits (WNL) through 2000 Hz sloping to a mild to moderately severe SNHL above was seen. The left ear had a mild low frequency SNHL rising to WNL through 4 KHz, sloping to a mild to moderate depression above. The speech reception thresholds (SRTs) agreed with pure-tone average (PTA); word recognition was very good in the right ear and excellent in the left ear. There was no rollover in the performance intensity function for phonetically balanced words (PIPB) in the right ear. Immittance findings indicated normal middle ear mobility in each ear with ipsilateral and contralateral acoustic reflexes present and no acoustic reflexes (AR) decay in either ear condition. The reliability was good. In brief, bilateral, asymmetric SNHL, right greater than left as shown in Figures 3-4 was found on the audiogram.

An magnetic resonance imaging (MRI) of the internal auditory canals was performed both before and after the administration of intravenous contrast, as per the standard department protocol. The internal auditory canals and cerebellopontine angles had normal size and configuration. The bilateral trigeminal nerves and cranial nerve VII and VIII complexes were normal. There were no abnormal enhancements or enhancing masses at the bilateral cerebellopontine angle cisterns or within the bilateral internal auditory canals. The inner ear structures including the semicircular canals, cochlea and vestibules were normal. A few of the right mastoid air cells were opacified with fluid. The left mastoid air cells were clear. The visualized brain was unremarkable. In brief, no masses in the internal auditory canals or cerebellopontine angles were seen.

We followed her closely during the evaluation period. Her vertigo continued to become less severe and less frequent; however, the tinnitus persisted 24/7. Her tinnitus was worse with exercise and turning her head to the right. She discontinued all her medications including gabapentin and Cymbalta for the past two weeks at the suggestion of the ear, nose, throat specialist (ENT), but no improvement occurred. She did not complain of otalgia, otorrhea, or ear blockage. This was followed by a detailed dental evaluation including the TMJ disease and no abnormalities were found. Considering that TMJ associated with her fibromyalgia could be a contributing cause, she also underwent massage therapy with no improvement, excluding this as a possible etiology. However, she confirmed the same pain for many years and has not worsened recently. Also, the massage therapy did not result in improvement in her symptoms. We recommended white noise therapy for tinnitus at nighttime or in quiet environments. Meditation has been shown to help with tinnitus, and we encouraged the patient to consider it. Unfortunately, her symptoms persisted. She refused to wear an auditory device and imatinib was not resumed. She was followed up in the oncology clinic every six weeks and her SNHL remained on her last visit one week ago.

## Discussion

Imatinib-induced ototoxicity such as SNHL has rarely been reported in patients with NSF and CML following the use of imatinib, but our case presents a patient who developed ototoxicity consisting of deafness, tinnitus, and vertigo secondary to the use of imatinib for GIST [4-5]. The previous authors presented a case of a 54-year-old woman with nephrogenic systemic fibrosis who developed bilateral fluctuating hearing loss one month after initiating imatinib. She experienced bilateral hearing loss within eight days after starting oral imatinib 400 mg daily. She was not on any concomitant medications or chemotherapeutic agents. Her dose was reduced to 200 mg daily, without any improvement in her hearing after two weeks and later the dose was escalated back to 400 mg daily again with no change. The audiogram revealed a bilateral SNHL. She also tried a five-day course of oral prednisone 60 mg daily with no improvement. In addition, despite stopping imatinib the repeat audiogram showed a flat bilateral SNHL [4]. The second case published previously reported a 19-year-old male with CML who developed bilateral irreversible sensorineural hearing loss after three months of imatinib. An audiometric evaluation confirmed the deafness [5].

There are two types of hearing losses associated with ototoxicity: SNHL refers to damage to the inner ear, including the cochlea and nerve pathways leading to the brain. SNHL is usually associated with chemotherapy medication and is oftentimes permanent and irreversible. Conductive hearing loss (CHL) refers to damage to the outer or middle ear, involving the tympanic membrane or ossicles. CHL is often associated with radiation therapy and can be temporary and reversible. There are many causes of deafness [6] but the most commonly used medicines that may cause SNHL are listed in Table 1.​

**Table 1:** Most commonly used medicines that may cause hearing loss List of the most commonly used medicines that may cause hearing loss

Drug Class	Examples	Comment
Salicylates	Aspirin	Especially when given in high doses (> 12 325-mg tablets of aspirin per day). A change in the membrane permeability of the outer hair cells is regarded as the underlying etiology. In addition, change in the cochlear blood supply as a result of the salicylate-induced imbalance of vasodilation by prostaglandins and/or vasoconstriction by leukotrienes may be a contributing factor.
NSAIDs	Ibuprofen Naproxen	Class effect
Aminoglycosides	Gentamicin Streptomycin Neomycin Vancomycin	Streptomycin is known to cause more damage to the vestibular portion than to the auditory portion of the inner ear. Neomycin is known to have the greatest cochleotoxic effect of all antibiotics. Gentamicin and tobramycin cause both vestibular and cochlear toxicity. Ototoxicity secondary to these antibiotics are most commonly encountered in people with concomitant kidney dysfunction or history of prior ear or hearing diseases. Ototoxic drugs should be avoided for otic topical application when the tympanic membrane is perforated. Similar to aminoglycosides in that it can be ototoxic when used intravenously in life-threatening infections
Loop diuretics	Furosemide (Lasix) Bumetanide	Usually found to be ototoxic when given intravenously for acute kidney failure, acute hypertensive crisis, or acute pulmonary edema/congestive heart failure, existing hearing deficits, or severe hypoalbinaemia. It is believed that loop diuretics damage the stria vascularis and/or the outer hair cells of the cochlea. It has also been noticed in studies that loop diuretics inhibit Na-K ATPase and adenyl cyclase in the stria. Concurrent administration of loop diuretics with aminoglycosides can exacerbate or cause aminoglycoside ototoxicity.
Chemotherapy		
Platinum Agents	Cisplatin Carboplatin Oxaliplatin	Platinum agents can cause both tinnitus and hearing loss. Exact etiology is unknown but hair cell damage, inhibition of Na+-K(ATPase) in the outer hair cells of the cochlea and atrophy of the stria vascularis are thought to be responsible. Hearing loss can occur either immediately after the first dose, or can be delayed until several months after completion of treatment. In general, hearing loss is profound and permanent, Also, sensorineural hearing loss occurs bilaterally, progresses decrementally, and is generally permanent.
Vinca alkaloid	Vincristine Vinblastine	Both agents have been noted to be associated with several cases of sensorineural hearing loss.
	Bleomycin	Possibly affecting discoidin domain receptor 1 (DDR1)
Quinine	Quinine and its synthetic substitutes	Quinine like aspirin can cause reversible SNHL associated with tinnitus and can occur in both healthy and malaria patients. Generally, SNHL is of rapid onset and resolves completely following withdrawal of the drug. It is suggested that these agents alter the membrane function of the outer hair cells especially in the region of the lateral cisternae.

Our patient was on none of the above drugs. There are many contributing factors that may affect the ototoxicity, such as dose, duration of therapy, concurrent renal failure, infusion rate, lifetime dose, co-administration with other drugs having ototoxic potential, and genetic susceptibility.

Imatinib is generally a well-tolerated drug. Imatinib is not an agent with associated otopathology. We added here the third case in total (all disease) and first case with GIST who developed sensorineural hearing loss as a consequence of imatinib. The exact etiology is not known. It is also possible that the exact incidence is under-reported based on comorbid conditions of the patient or gradual use in previous studies might have only captured Grade III and IV. Because mutations in c-Kit constitute the intended molecular target for imatinib in the treatment of GIST, it is possible that the pharmacologic inhibition of c-Kit might negatively affect hearing, probably a neurotoxic effect on the auditory nerve [7-10]. Future studies of the effect of imatinib and/or TK inhibition on the inner ear as a possible cause of imatinib-related ototoxicity are indicated. Patients treated with imatinib should be followed closely for early recognition of sensorineural hearing loss, and if hearing loss is noticed, then imatinib should be discontinued immediately. Moreover, oncologists and other physicians should report similar cases as we have to enhance the incidence and management.

## Conclusions

In conclusion, imatinib should be included in the differential of vestibular and auditory toxicity, especially SNHL seen in patients while receiving imatinib. Hence, careful attention and appropriate clinical evaluation are required for patients presenting with such symptoms during imatinib treatment. Physicians caring for patients receiving imatinib should be aware of this potential toxicity. Hearing loss prevention and treatment during chemotherapy for cancer needs further research to identify any risk factors, including genetic, and develop otoprotective agents or find new strategies to optimize old agents.
